# Effect of Nicotinamide Against *Candida albicans*

**DOI:** 10.3389/fmicb.2019.00595

**Published:** 2019-03-26

**Authors:** XinRui Xing, ZeBin Liao, Fei Tan, ZhenYu Zhu, Yuanying Jiang, YingYing Cao

**Affiliations:** ^1^Shanghai Skin Disease Hospital, Tongji University School of Medicine, Shanghai, China; ^2^School of Pharmacy, Second Military Medical University, Shanghai, China; ^3^Department of Radiation Medicine, Faculty of Naval Medicine, Second Military Medical University, Shanghai, China; ^4^Department of Pharmacology, Shanghai Tenth People’s Hospital, Tongji University School of Medicine, Shanghai, China

**Keywords:** nicotinamide, *Candida albicans*, drug resistance, fluconazole, GIN4

## Abstract

Nicotinamide (NAM) has a long history in clinical applications and can be safely used for treating various diseases. In recent years, NAM was found to exhibit antimicrobial activities, inhibiting the growth of *Plasmodium falciparum*, *Mycobacterium tuberculosis*, and human immunodeficiency virus (HIV). Here we investigated the activity of NAM against *Candida albicans*, one of the most prevalent human fungal pathogens. Our results showed that NAM exhibited significant antifungal activity against *C. albicans*, including fluconazole-resistant isolates. NAM could also effectively suppress biofilm formation. In addition, NAM exhibited antifungal activity against non-*Candida albicans* species and *Cryptococcus neoformans*. Combination of NAM and fluconazole showed an even strong antifungal activity. The antifungal activity of NAM was further confirmed in a mouse model of disseminated candidiasis. Confocal laser scanning microscopy revealed that NAM increased cell wall β-glucans exposure and chitin content while decreased mannan level. Furthermore, by screening the *C. albicans* homozygous deletion mutant library, the *C. albicans* mutant lacking GIN4, which encodes a septin regulatory protein kinase and is essential for the maintenance of cell wall integrity, was identified to be high sensitive to NAM. These findings suggested that NAM might exhibit antifungal activities through affecting cell wall organization.

## Introduction

In recent years, with the increase in the number of immunocompromised individuals such as organ transplant recipients, AIDS patients, and patients receiving chemotherapy, a rise in fungal infection has been observed. *Candida albicans* is one of the most prevalent human fungal pathogens, causing superficial mycoses, invasive mucosal infections and disseminated systemic disease (Ng et al., 2015). The mortality related to *C. albicans* bloodstream infections is usually estimated to be approximately 40% (Wisplinghoff et al., 2014). The antifungal agents commonly used in clinical practice mainly include azoles, echinocandins and polyenes. However, there are various therapeutic limitations exist in these antifungal drugs. For example, the broad utilization of fluconazole has led to rapidly emerging drug-resistant isolates. Thus, development of safer and more effective antifungal drugs remains a formidable challenge.

Nicotinamide (NAM), an amide form of vitamin B3, has been known as a safe agent and can be used at high doses for various therapeutic applications (Ma and Medenica, 1983; Handfield-Jones et al., 1988; Libri et al., 2014). NAM is safe even administered at a high dosage of 6 g/day in human (Knip et al., 2000). NAM showed the potential to cure pellagra and potentially treating mitochondrial encephalopathies and neurodegenerative diseases (Beal et al., 1994; Chen and Damian, 2014). The phase III clinical trials showed that NAM was useful in the prevention of skin cancer (Chen et al., 2015). In recent years, it has been shown that NAM executes antimicrobial activities, inhibits cell proliferation, and enhances the antiproliferative effect of cytostatic drugs. It was shown that NAM can inhibit growth of *Plasmodium falciparum*, *Mycobacterium tuberculosis* and human immunodeficiency virus (HIV) (Murray, 2003; Sereno et al., 2005; Prusty et al., 2008; Unciti-Broceta et al., 2013; Wang et al., 2013; Tcherniuk et al., 2017). In this study, we evaluated the antifungal effect of NAM *in vitro* and *in vivo* and explored the underlying mechanisms.

## Materials and Methods

### Strains, Cultures, and Chemicals

*C. albicans* strains SC5314 was a generous gift obtained from Professor Dominique Sanglard (Centre Hospitalier Universitaire Vaudois, Lausanne, Switzerland). All clinical isolates are provided by Changhai Hospital of Shanghai, China. The *C. albicans* homozygous deletion strains were obtained from the Fungal Genetics Stock Center (Kansas State University, 4024 Throckmorton Plant Sciences Center, Manhattan, KS 66506, United States). Strains were routinely maintained on Sabouraud dextrose agar (1% w/v peptone, 4% w/v dextrose, and 1.8% w/v agar) and grown in YPD liquid medium (1% yeast extract, 2% peptone, and 2% dextrose) at in an orbital shaker at 30°C. RPMI-1640 medium were purchased from Gibco (United States). Nicotinamide (NAM) and fluconazole were purchased from Sigma-Aldrich Co. LLC (St. Louis, MO, United States). For all the experiments, 6.4 mg/ml FCZ and 2M NAM in ultra-pure distilled water were used as stocks, and added to the culture suspensions to obtain the required concentrations.

### Antifungal Susceptibility Test

Antifungal susceptibility testing was performed in 96-well tissue culture plates (Corning Inc., Corning, NY, United States) using a broth microdilution protocol of the Clinical and Laboratory Standards Institute M27-A3 method, with a few modifications (Quan et al., 2006). In brief, the initial concentration of fungal cells in RPMI-1640 medium was 10^3^ CFU/ml, and the final concentrations ranged from 1.25 to 320 mM for NAM and 0.125–64 μg/ml for fluconazole. Plates were incubated at 35°C for 24 h. MIC_50_ was determined as the lowest concentration of the drugs (alone or in combination) that inhibited growth by 50%.

### Time-Kill Curve Assay

Time-kill curve assay was performed as described previously (Li et al., 2013). Briefly, exponentially growing *C. albicans* cells grown to exponential phase were washed with phosphate-buffered saline (PBS) and resuspended in RPMI-1640 medium to 1 × 10^3^ cells/ml. Then different concentrations of NAM were added to the cell suspensions. The samples were incubated with agitation at 30°C. At each designated time points (0, 3, 6, 9, and 12 h), portions of cell suspensions were withdrawn and plated on YPD agar in serial dilutions to determine the CFU/ml of the cell suspensions.

### Biofilm Formation Assay

The *in vitro* biofilm formation assay was carried out as described previously (Ramage et al., 2001). In brief, *C. albicans* SC5314 cells (5 × 10^5^ cells/ml) in RPMI-1640 medium were added to a 96-well tissue culture plate (Corning Inc., Corning, NY, United States) for 90 min of adhesion at 37°C. Then non-adherent cells were removed and fresh medium containing different concentrations of drugs was added. The plate was incubated at 37°C for 24 h. The growth of biofilms was measured with a 2,3-bis-(2-methoxy-4-nitro- 5-sulfophenyl)-2H-tetrazolium-5-carboxanilide (XTT) reduction assay, a reaction catalyzed by mitochondrial dehydrogenases (Ramage et al., 2002). In brief, biofilm cells were washed with PBS and then incubated with 0.5 mg/ml of XTT and 1 mM of menadione in PBS at 37°C for 90 min. The optical density (OD) was determined at 490 nm using a microtiter plate reader.

### Mouse Model of Disseminated *Candidiasis*

Animal experiments were approved by the Animal Ethics Committee of the Second Military Medical University (Shanghai, China). Six week-old female BALB/c mice (Sino-British SIPPR/BK Lab Animal, Shanghai, China) were injected intravenously with 5 × 10^6^ cells of the *C. albicans* SC5314 in 0.2 ml of sterile saline (0 day). NAM was administered intraperitoneally at 0, 1, 2, 4, and 6 day. Sterile saline solution was employed as control. The mortality of the mouse was monitored daily.

For determination of histology, mice were sacrificed after 6 days of infection. The kidneys were fixed in10% formalin, embedded in paraffin wax, and sectioned longitudinally. Specimens were stained with hematoxylin and eosin (H&E) and with periodic acid-Schiff (PAS) staining for the assessment of fungal invasion.

### Cell Wall Components Assay by Confocal Laser Scanning Microscopy

*C. albicans* SC5314 cells treated with 20 mM of NAM for 16 h were washed with PBS buffer for the determination of cell wall components. To analyze β-(1,3)-glucan of cell wall, cells were incubated with anti-β-(1,3)-glucan antibody (Biosupplies Inc.) overnight at 4°C and then stained by 10 μg/ml Cy3-labeled antibody for 1 h at 30°C. The α-mannopyranosyl and chitin were stained by 50 μg/ml Concanavalin A (Thermo Fisher Scientific) and 30 μg/ml calcofluor white (Sigma-Aldrich) for 1 h, respectively. After being stained, cells were washed with PBS, diluted into 1 × 10^6^ cells/ml, scanned by confocal laser scanning microscope (TCS SP5; Leica) and then analyzed by LAS AF Lite program.

### Cellular Surface Hydrophobicity (CSH) Assay

Cellular surface hydrophobicity (CSH) was determined by a water-hydrocarbon two-phase assay as described previously (Klotz et al., 1985). In brief, *C. albicans* SC5314 cells treated with different concentrations of NAM for 16 h were washed with PBS and resuspended in YPD medium (OD_600_ = 1.0). Then a total of 1.2 ml of the cell suspension was pipetted into a clean glass tube and overlaid with 0.75 ml of cyclohexane. The mixture was vortexed for 3 min and then stood at room temperature for phase separation. Soon after the two phases had separated, the OD_600_ of the aqueous phase was determined, and the OD_600_ for the group without the cyclohexane overlay was used as the control. Relative CSH was calculated as follows: [(OD_600_ of the control - OD_600_ after cyclohexane overlay)/OD_600_of the control] × 100.

### Screening of *C. albicans* Cell Wall Mutant Library for NAM Susceptibility

The *C. albicans* homozygous deletion mutant library was obtained from the Fungal Genetics Stock Center, among which 42 cell wall-related mutants and the corresponding wild type strains were chosen for NAM susceptibility test (Homann et al., 2009; Nobile and Mitchell, 2009; Noble et al., 2010). The final concentrations used for NAM ranged from 1.25 to 320 mM. Each screen was performed in duplicate. Relative growth as monitored by OD_600_ was normalized to untreated control for each strain and is displayed in heat map format. The screening output was validated by retesting the NAM sensitivities of a selection of mutants using drop tests. To achieve this, strains grown overnight in YPD were serially diluted, dropped on to YPD solid media containing different concentrations of NAM, and then grown at 30°C for 2 days.

### Statistical Criteria

Statistics were calculated in GraphPad Prism 6.0 (GraphPad Software, San Diego, CA, United States), in which *P*-value of < 0.05, < 0.01, or < 0.001 was considered statistically significant.

## Results

### Effect of NAM on *C. albicans* Growth

**FIGURE 1:**
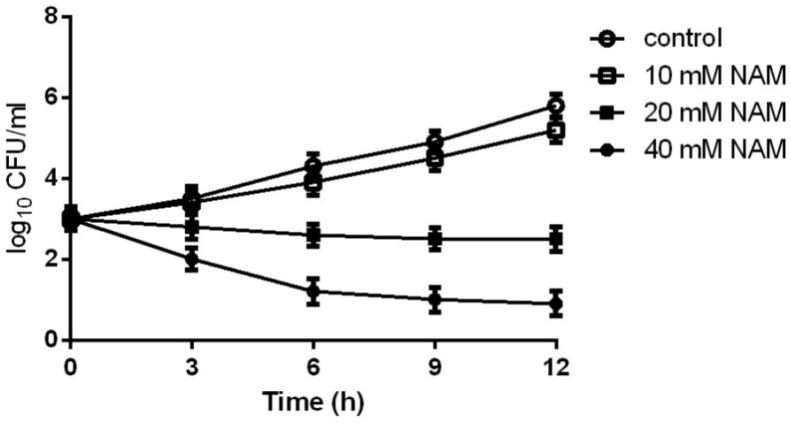
Time-kill curves of NAM against *C. albicans*. Different concentrations of NAM were added to 1 × 10^3^ cells/ml of *C. albicans* SC5314 cells suspended in RPMI 1640 medium. At each designated time points, portions of cell suspensions were withdrawn and plated on YPD agar to determine the CFU/ml of the cell suspensions. The data shown are means ± standard deviations from three independent experiments.

We first evaluated the inhibitory effect of NAM on *C. albicans* SC5314, a standard strain widely used in the studies of *C. albicans*. As shown in Table 1, the MIC_50_ of NAM against *C. albicans* SC5314 was 20 mM. Time-kill curve assay showed that NAM at the concentration of 10 mM had a slight inhibitory effect on *C. albicans*. However, addition of 20 mM NAM resulted in an obvious antifungal activity, as approximately 3 log10 CFU/ml decrease was observed as compared to the control group after 12 h of treatment. Treatment of *C. albicans* cells with 40 mM NAM led to an even strong antifungal activity, with a remarkable drop of the survival curve as compared to the untreated group (Figure 1).

**Table 1:** Effects of nicontinamide used alone or in combination with fluconazole against fungi.

Strains	MIC_50_ alone^a^		MIC_50_ in combination	
	NAM	Fluconazole	NAM	Fluconzole
***Candida albicans***				
SC5314	20	0.25	10	0.0625
Y0109	20	0.25	10	0.0625
638	20	16	5	1
647	40	32	10	0.5
663	20	64	5	0.5
***Candida parapsilosis***				
561	20	2	10	1
567	40	1	10	0.25
582	40	2	5	0.125
***Candida tropicalis***				
467	40	4	20	1
411	40	2	5	0.5
489	40	4	10	0.5
***Candida glabrata***				
773	40	64	10	2
791	20	32	5	0.5
741	20	64	5	2
***Candida krusei***				
213	20	64	5	2
204	20	64	5	1
266	40	64	5	2
***Cryptococcus neoformans***				
73046	40	64	10	4
73509	80	64	20	4
4755	20	8	5	0.5
4794	40	8	5	0.5
4233	40	4	5	1
4205	40	8	5	0.5

*^a^The unit of nicotinamide is mM and the units of Fluconazole is μg/ml.*

Since azole-resistant isolates occur frequently, we investigated the activity of NAM against clinically fluconazole-resistant C. a*lbicans* isolates 638, 647, and 663. As shown in Table 1, NAM exhibited similar inhibitory activity against these fluconazole-resistant *C. albicans* isolates as the fluconazole-sensitive SC5314 strain. Moreover, the presence of NAM remarkably enhanced the activities of fluconazole against the fluconazole-resistant *C. albicans*. Specifically, the MIC_50_ of NAM and fluconazole used alone against clinical isolate 663 was 20 mM and 64 μg/ml, respectively. Combined used of NAM and fluconazole resulted in the MIC_50_ of 5 mM and 0.5 μg/ml, respectively.

### Antifungal Activity of NAM on Other Pathogenic Fungi

These inhibitory activity of NAM against *C. albicans* prompted us to test whether other clinically relevant fungal isolates were susceptible to NAM. So we next investigated the activity of NAM against several clinical isolates, including *Candida parapsilosis, Candida tropicalis, Candida glabrata, Candida krusei*, and *Cryptococcus neoformans*. We observed that NAM showed inhibitory activities against all these isolates tested, with the MIC_50_ ranging from 20 to 80 mM. It should be noted that *Candida glabrata* and *Candida krusei* has been reported to be inherently less susceptible to fluconazole (Beardsley et al., 2018). As expected, these strains tested here displayed high resistance to fluconazole (MIC_50_γ 32 μg/ml). Combination of NAM and fluconazole showed an even strong effect against the *Candida glabrata* and *Candida krusei* isolates and remarkably reduced the MIC of these isolates to fluconazole. Addition of NAM could also remarkably enhance the activity of fluconazole against *Cryptococcus neoformans* (Table 1). These results indicated broad antifungal properties for NAM.

### NAM Inhibits *C. albicans* Biofilm Formation

**FIGURE 2:**
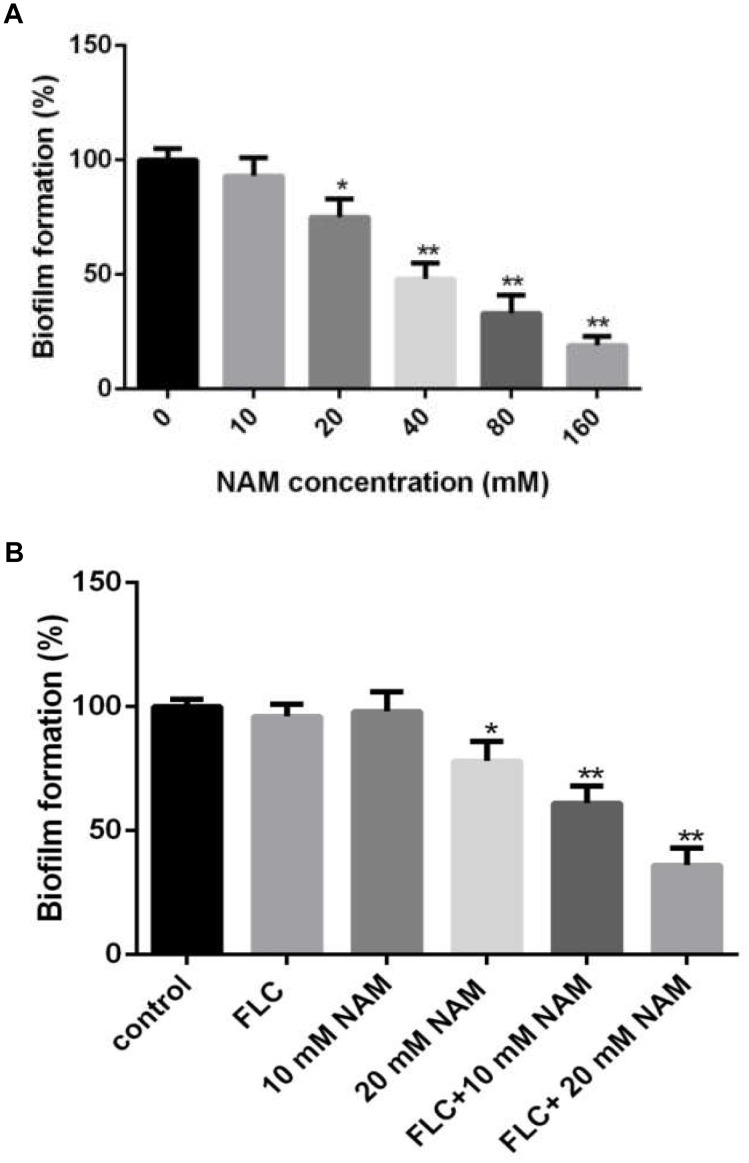
**(A)** Effect of different concentrations of NAM on *C. albicans* biofilm formation. **(B)** *C. albicans* biofilms formation in the presence of 16 μg/ml fluconazole (FLC) and different concentrations of NAM. Biofilm formation was evaluated using an XTT reduction assay, and the results are presented as the percent of drug-treated biofilms relative to the control (drug-free) biofilms. The data shown are means ± standard deviations from three independent experiments. ^∗^*P* < 0.05; ^∗∗^*P* < 0.01 as compared to the control group.

Since biofilm formation is an important factor for the pathogenesis of *C. albicans*, leading to high resistance of this pathogen to a wide range of antifungals (Finkel and Mitchell, 2011), here we examined the effect of NAM on biofilm formation of *C. albicans*. XTT reduction assays revealed that NAM showed inhibitory effect on biofilm formation (Figure 2A). More specifically, 20 mM NAM inhibited *C. albicans* biofilm by approximately 25%, and the inhibitory activity on biofilm was enhanced when the concentration of NAM used was increased. Addition of 40 mM NAM inhibited biofilm formation by 52%, while over 80% percent of the biofilm was inhibited in the presence of 160 mM NAM. Moreover, the synergism of NAM and fluconazole against *C. albicans* biofilm was observed. As shown in Figure 2B, addition of 10 mM or 16 μg/ml fluconazole alone did not exhibit an significant impact on biofilm formation. However, the combination of these two agents resulted in a dramatic reduction in biofilm formation.

### Antifungal Activity of NAM in a Mouse Model of Disseminated *Candidiasis*

**FIGURE 3:**
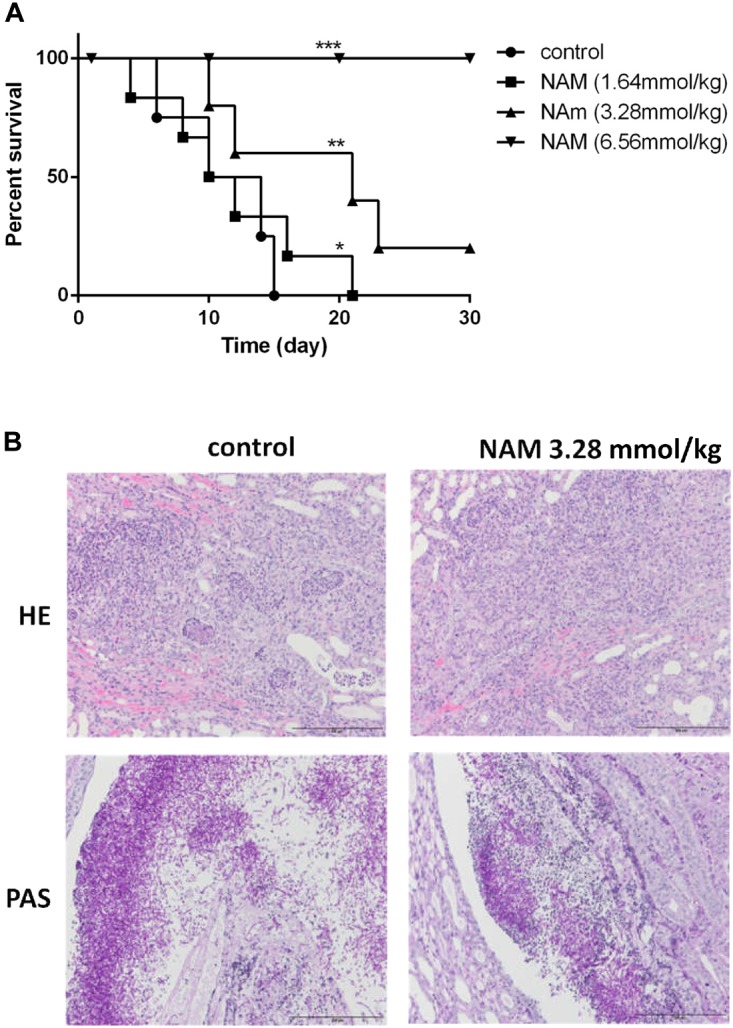
**(A)** Survival curves of BALB/c mice with disseminated *candidiasis* treated by NAM. The mice were infected with 5 × 10^6^ CFU *C. albicans* SC5314 in 200 μl sterile saline intravenously and monitored for 30 days (*n* = 8 per group). **(B)** Inflammatory responses and *C. albicans* colonization in mouse kidney (×200 magnification). Representative HE (hematoxylin and eosin) and PAS (periodic acid-Schiff) staining of kidneys from BALB/c mice infected with 5 × 10^6^ CFU *C. albicans* SC5314 at day 6 were used to detect the inflammatory cells influx and the extent of tissue necrosis or hyphae invasion. ^∗^*P* < 0.05; ^∗∗^*P* < 0.01, ^∗∗∗^*P* < 0.0005 as compared to the control group.

We further evaluated the antifungal activity of NAM in the mouse model of disseminated *candidiasis*. Although treatment of 1.64 mmol/kg of NAM had a slight impact on the mouse mortality rate as compared to the control (saline-treated) group, 3.28 mmol/kg of NAM-treated group showed a significantly prolonged survival period as compared to the control group. It should be noted that NAM at the dose of 6.56 mmol/kg could absolutely protect the mouse from *C. albicans* infection, with 100% survival rate (Figure 3A).

To investigate the effect of NAM on tissue damage and fungal invasion, the kidneys were sectioned longitudinally to have an overview of renal hilum, renal cortex and renal medulla. As is shown in Figure 3B, a large area of necrosis, neutrophil infiltration and hyphae invasion in renal cortex, renal medulla and especially the area near the renal pelvis was evident in the kidney from untreated group. Treatment of 3.28 mmol/kg of NAM remarkably relieved the degree of pathological change, in which much less necrosis and neutrophil infiltration of renal medulla or tissue near the renal pelvis were observed.

### NAM Influences *C. albicans* Cell Wall Organization

**FIGURE 4:**
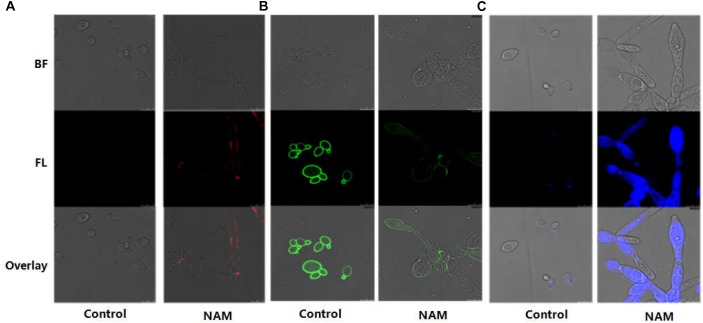
Effect of NAM on cell wall organization. *C. albicans* cells were treated with 20 mM of NAM for 16 h and then stained with β-glucan antibody to detect β-(1,3)-glucan **(A)** Concanavalin-A to detect mannan **(B)** and calcofluor white to detect chitin **(C)**. Bright field (BF), fluorescence (FL), and overlay were shown individually.

In *C. albicans*, cell wall is mainly composed of an inner layer of chitin, a middle layer of β-glucan and an outer fibrillar layer of mannan. β-glucan is normally masked by a dense layer of glycosylated proteins. We first investigated the impact of NAM on β-glucan exposure by staining of the cell wall with a monoclonal β-1,3-glucan antibody. As shown with confocal laser scanning microscopy, no fluorescence signal was detected in the untreated cells because of the inner location of this polysaccharides. Treatment with NAM resulted in a significant increase in fluorescence signal around the cell wall periphery, indicating the exposure of β-glucans on the external surface of the *C. albicans* cells (Figure 4A). We next examined the effect of NAM on mannan by staining the cells with ConA. As shown in Figure 4B, a remarkably decreased fluorescence intensity in NAM-treated cells was observed, indicating lower levels of mannan present in the cell wall as compared to the untreated cells. Meanwhile, Calcofluor White (CFW) staining revealed that the content of chitin was elevated upon NAM treatment (Figure 4C). It should be noted that the *C. albicans* cells in all NAM-treated groups displayed abnormal morphology, striking with enlarged cells and pseudohyphae formation as compared to the untreated cells. Taken together, these results implicated that NAM might exhibit antifungal activities through influencing cell wall organization.

### NAM Reduces Cellular Surface Hydrophobicity

**FIGURE 5:**
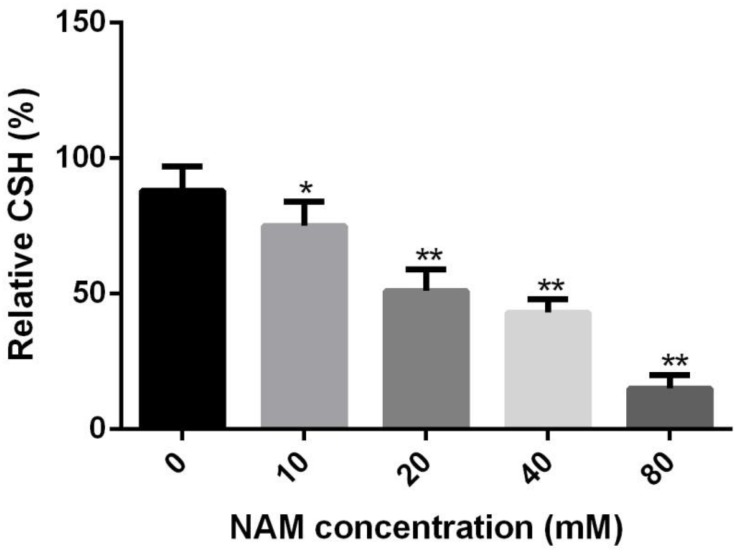
Effects of NAM on the cell surface hydrophobicity (CSH). *C. albicans* cells were treated with different concentrations of NAM for 16 h and then CSH was estimated by the water-hydrocarbon two-phase assay. Data are expressed as the mean ± standard deviation of the independent assays in triplicate. ^∗^*P* < 0.05; ^∗∗^*P* < 0.01 as compared to the NAM-free group.

Since cellular surface hydrophobicity (CSH), a important indicator of adhesion and biofilm formation ability, is correlated with cell wall composition (Raut et al., 2010), we determined the CSH of *C. albicans* upon NAM treatment. As shown in Figure 5, a negative correlation was observed between NAM concentrations and the CSH of *C. albicans* cells. The relative CSH of untreated *C. albicans* cells was high (about 88%). Addition of NAM could decrease CSH in a dose-dependent manner. When the *C. albicans* cells was treated with 20 mM of NAM, the relative CSH was reduced to approximately 51%. Addition of 40 mM of NAM led to an almost twofold decrease in CSH compared to the untreated *C. albicans* cells.

### Genetic Screening Reveals That GIN4 Is Responsible for NAM Susceptibility

**FIGURE 6:**
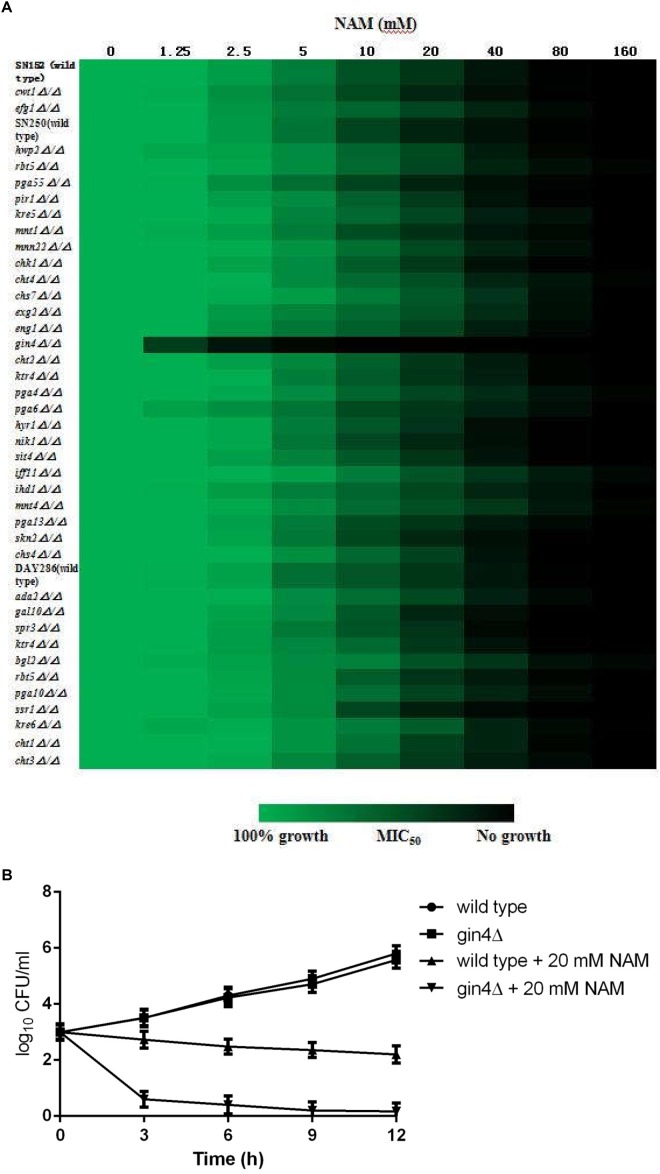
**(A)** Sensitivity of *C. albicans* mutants to NAM. Antifungal susceptibility testing with NAM was performed using wild type and *C. albicans* mutant strains. Relative growth as monitored by OD_600_ was normalized to the drug-free control for each strain and is displayed in heat map format. Data represent the mean of results from three independent experiments. **(B)** Time-kill curves of NAM against *C. albicans gin4Δ/Δ* mutant and wild type strain SN250. 1 × 10^3^ cells/ml of *C. albicans* cells suspended in RPMI 1640 medium were treated with 20 mM of NAM and then incubated with agitation at 30°C. At each designated time points, portions of cell suspensions were withdrawn and plated on YPD agar to determine the CFU/ml of the cell suspensions. The data shown are means ± standard deviations from three independent experiments.

In order to further probe the antifungal mechanism of NAM, we screened a *C. albicans* homozygous deletion mutant library for growth in the presence of NAM. In view of the impact of NAM on cell wall, our screening of NAM sensitivity focused on the mutants in which the genes involved in cell wall synthesis and remodeling were deleted. Among the 42 strains tested, a mutant lacking GIN4, which encodes a septin regulatory protein kinase, was identified to be most susceptible to NAM treatment as compared to wide type strain (Figure 6A). The Susceptibility of *gin4Δ/Δ* mutant to NAM was further confirmed by time–kill curves (Figure 6B). In the absence of NAM, there were no significant difference in the growth between the wild type and *gin4Δ/Δ* mutant. However, when 20 mM of NAM was added, the survived cells of *gin4Δ/Δ*mutant were dramatically reduced, yielding approximately >2 log10 CFU/ml decrease as compared with the wild type strain. These results suggested that GIN4 played an important role in the antifungal activity of NAM.

## Discussion

In recent years, with the increase of patients with impaired immune function, pathogenic fungi pose a rapidly increasing threat to human health. *C. albicans* is one of the most frequently isolated fungal pathogen in humans. Although there is an urgent need for efficient antifungal therapy, highly effective antifungal agents available are still limited. In this study, we demonstrated the antifungal activity of NAM, a cheap and safe vitamin with long history of clinical application. NAM showed inhibitory effect on *C. albicans*, including fluconazole-resistant clinical isolates. It is known to us all that fluconazole-resistant *C. albicans* isolates are emerging rapidly due to the widely used of this agent. Here NAM exerted inhibitory activity against fluconazole-resistant *C. albicans* isolates at the similar concentrations as the fluconazole-sensitive strains. Moreover, NAM could strongly enhance the activity of fluconazole against fluconazole-resistant *C. albicans*. When used in combination, the MIC_50_ for fluconazole could be reduced by the maximum of 128-fold (from 64 to 0.5 μg/ml). Besides, NAM displayed broad-spectrum activity against multiple clinical isolates of *Candida* spp. and *Cryptococcus neoformans*. Thus, the clinical application of NAM opens a great possibility, which could overcome the problem of drug resistance and minimize adverse effects of currently used antifungals.

Biofilms are heterogeneous microbial communities consist of cells adhering to medical devices or human organs. One of the major contributions to the virulence of *C. albicans* is its ability to form biofilms which show high resistance to several commonly used antifungal agents, including fluconazole and amphotericin B (Chandra et al., 2001). Our study showed that NAM could not only exert antifungal effect on planktonic *C. albicans* cells, but also display inhibitory activity on biofilm formation. Since fluconazole cannot efficiently inhibit biofilm formation, we tested the impact of NAM on the antibiofilm activity of fluconazole and found that NAM could significantly enhance the activity of fluconazole against *C. albicans* biofilm. These results suggested that NAM could be a promising agent in eradicating *C. albicans* biofilms.

In fungi, cell wall is a crucial extracellular organelle that protects the cell from lysis during environmental stress. The essential role of cell wall has been highlighted by the success of the echinocandins in treating fungal infection (Odds et al., 2003). *C. albicans* cell wall, in addition to its function in maintaining cellular viability, plays an important role in pathogenesis (Gow et al., 2007). Mannan, β-glucan and chitin are the three main structural components of *C. albicans* cell wall. These various cell wall components are efficiently regulated in concert to cell growth. Here treatment of NAM led to the decrease of mannan level on the outer layer of cell wall. Moreover, an increased level of chitin, a component in the inner layer of cell wall, was observed upon NAM treatment. This result was consistent with previous conclusion that agents affecting a component of the fungal cell wall often led to the modification of other cell wall components in compensation to help maintain cell wall strength and integrity (Garcia et al., 2004). For example, upregulation of chitin level was an adaptive response of *C. albicans* upon echinocandins treatment (Walker et al., 2008). In addition, we found that NAM could unmask the underlying β-glucan in the middle layer of cell wall. This result was similar with the effect of caspofungin, a well-known cell wall-targeting antifungal agent belonged to echinocandins. Taken together, NAM might kill fungal cells through affecting cell wall.

In view of the impact of NAM on cell wall, we screened a collection of *C. albicans* mutants, in which the genes involved in cell wall remodeling and synthesis were deleted, for NAM sensitivity. Our results showed that the *C. albicans* mutant lacking GIN4 displayed much higher sensitivity to NAM as compared to the wild type strain, indicating an important role of this gene in the antifungal activity of NAM. GIN4 encodes a septin regulatory protein kinase and is known to phosphorylate septins, which are cytoskeletal proteins and, besides their primary role as scaffolds for cytokinesis, are essential for the maintenance of cell wall integrity (Altman and Kellogg, 1997; Mortensen et al., 2002; Li et al., 2012; Au Yong et al., 2016). Previous studies reported that *C. albicans gin4Δ/Δ* mutant were characterized by extreme bud elongation, constitutively formed pseudohyphae and were unable to form hyphae in the presence of serum (Wightman et al., 2004; Li et al., 2012). This phenotype is similar with the anomalous filamentous growth of *C. albicans* cells upon NAM treatment showed in this study. In *Saccharomyces cerevisiae*, septins are required for localization of chitin synthase Chs3. Septin-defective mutants display aberrant sites of chitin deposition (Roh et al., 2002). In baker’s yeast, genetic deletion of GIN4 resulted in altered phosphorylation and mislocalization of Chs4, which indirectly changes chitin levels in the cell wall (Gohlke et al., 2018). Badrane et al. (2016) reported that GIN4 and septin localization is essential for maintaining *C. albicans* cell wall integrity in response to caspofungin. Consistently, a *gin4Δ/Δ* mutant is hypersensitive to capofungin. In our study, elevated chitin content, which functions to in provide protection to stressed cells by reinforcing the cell wall, was observed in the wide type cells upon NAM treatment. Since septins are regulated by GIN4, we speculated that *gin4Δ/Δ* mutant could not efficiently remodel cell wall by increasing chitin content due to the lack of normal septin function, thus displaying high sensitivity to NAM treatment.

## Conclusion

In conclusion, the results presented in this paper indicate that NAM has significant antifungal activity and may be of potential interest in the clinical application.

## Author Contributions

YC and YJ conceived and designed the experiments. XX, ZL, and FT performed the experiments. XX, ZZ and YC analyzed the data. YC and ZL wrote the manuscript.

## Conflict of Interest Statement

The authors declare that the research was conducted in the absence of any commercial or financial relationships that could be construed as a potential conflict of interest.
